# Acute rupture of a sinus of Valsalva aneurysm into the right atrium: a case report and a narrative review

**DOI:** 10.1186/s12872-020-01383-7

**Published:** 2020-02-18

**Authors:** Ata Doost, Julie-Ann Craig, Siang Yong Soh

**Affiliations:** 1 Department of Cardiology, Royal Perth Hospital, Perth, Western Australia 6000 Australia; 2 Department of Cardiology, Calvary Public Hospital, Bruce, Australian Capital Territory 2617 Australia

**Keywords:** Sinus of Valsalva, Aneurysm, Aortic aneurysm, Congenital heart disease

## Abstract

**Background:**

Sinus of Valsalva aneurysm (SVA) is a rare cardiac anomaly which has potential for spontaneous rupture into other cardiac chambers or the pericardial space (depending on its location). A ruptured SVA has a very poor prognosis with high morbidity and mortality. The development of a shunt between the sinus of Valsalva and right-sided cardiac chambers results in a continuous murmur on examination. Our case report is a case of SVA rupture into the right atrium.

**Case presentation:**

In this case report, we describe a 23-year-old patient with an acute onset of chest pain, shortness of breath, palpitations and dizziness starting 2 days prior to presentation to the emergency department. The patient was initially treated for presumed pulmonary embolism overnight while awaiting CTPA the next morning. However, further examination by the inpatient medical team demonstrated a continuous machinery cardiac murmur. Subsequent echocardiography demonstrated an acutely ruptured SVA with shunting to the right atrium. Emergency surgical repair resulted in an excellent outcome for the patient.

**Conclusion:**

A thorough clinical history and physical examination is the cornerstone of all medical encounters. An SVA could be asymptomatic until acute rupture. Echocardiography is the preferred initial diagnostic tool. Additional imaging techniques can be used to confirm the diagnosis. In cases of rupture, prognosis is poor and surgical repair is always required.

## Background

A sinus of Valsalva aneurysm (SVA) is a rare cardiac anomaly which could be congenital or acquired. Aneurysms of the sinus of Valsalva are typically more common in men (4:1) and there is a higher incidence in Asian populations [1]. An SVA is a consequence of weakness of the elastic lamina at the junction of the aortic media and the annulus fibrosis [2]. Here, we present a case of a 23-year-old woman with an acute rupture of an SVA into the right atrium with significant left-to-right shunting.

## Case presentation

A 23-year-old female Chinese university student presented to the emergency department with acute onset dyspnoea, chest pain, palpitations and dizziness for 2 days. Her vital signs included a blood pressure of 105/79 mmHg with a pulse rate of 110 beats per minute, a respiratory rate of 16 per minute and oxygen saturation of 94% on room air. Initially she was treated in ED with full dose anti-coagulation for suspected pulmonary embolism overnight while awaiting CTPA the next morning. Further examination demonstrated an incidental finding of a continuous machinery cardiac murmur best heard at the left parasternal border, loudest in systole (5/6), and 3/6 in diastole with a palpable thrill across the precordium. There was no relevant past medical history or significant family history. Her medical check-up performed during her application for an Australian student visa was unremarkable.

Electrocardiogram (ECG) showed sinus tachycardia. Laboratory results demonstrated negative high sensitivity troponin I and positive D-Dimer (1.12 mg/LF). Computed tomography pulmonary angiography (CTPA) was negative for pulmonary embolism, with no other abnormalities noted.

**Fig. 1:**
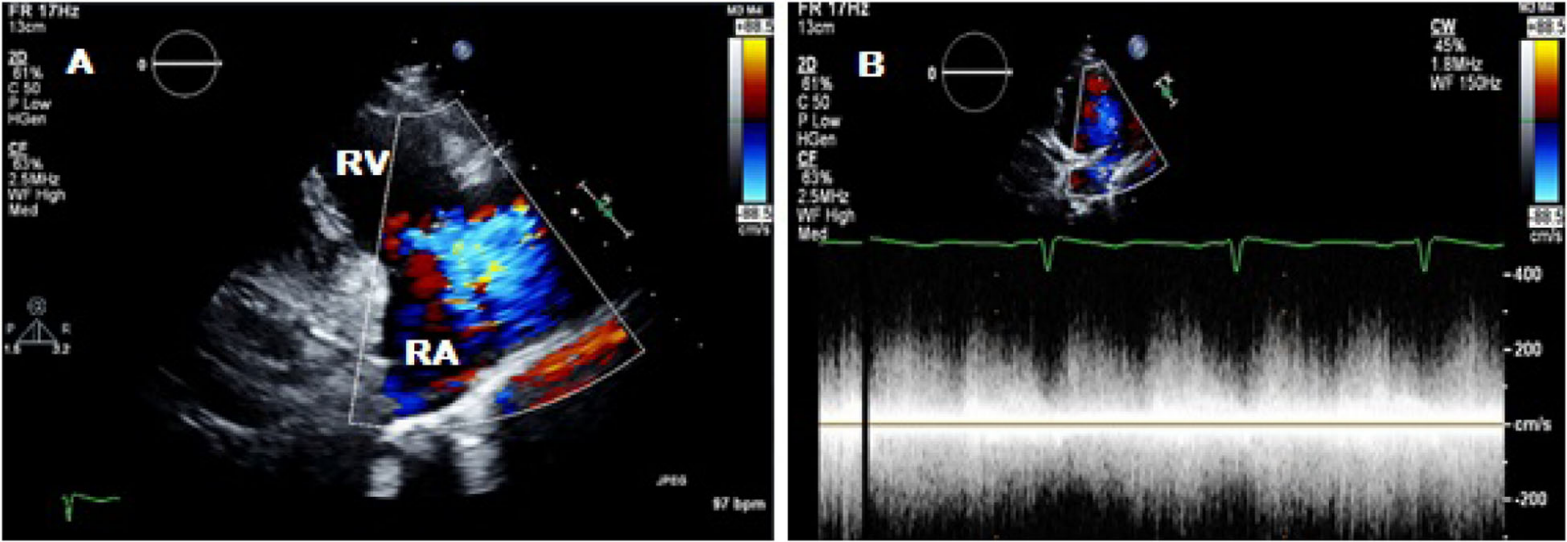
Two-dimensional transthoracic echocardiography in parasternal long axis right ventricular inflow view; **a** tricuspid valve regurgitation jet on colour Doppler echocardiography. **b** Continuous wave Doppler through tricuspid valve regurgitation jet showing a continuous flow. RV, right ventricle; RA, right atrium

**Fig. 2:**
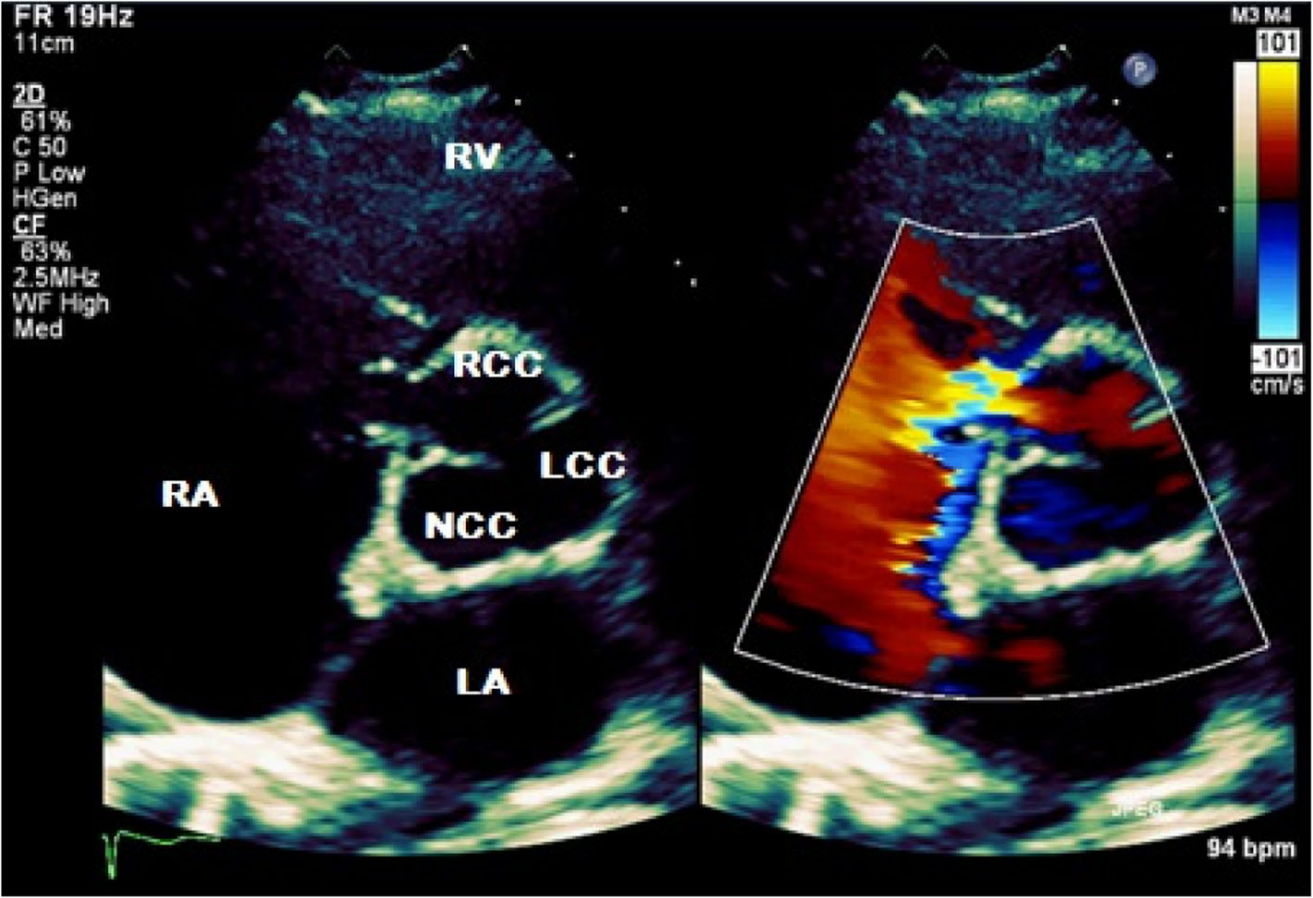
Two-dimensional transthoracic echocardiography in parasternal short axis with colour compare view demonstrating a shunt from the right coronary sinus to the right atrium. RV, right ventricle; RA, right atrium; LA, left atrium; RCC, right coronary cusp; NCC, non-coronary cusp; LCC, left coronary cusp

**Fig. 3:**
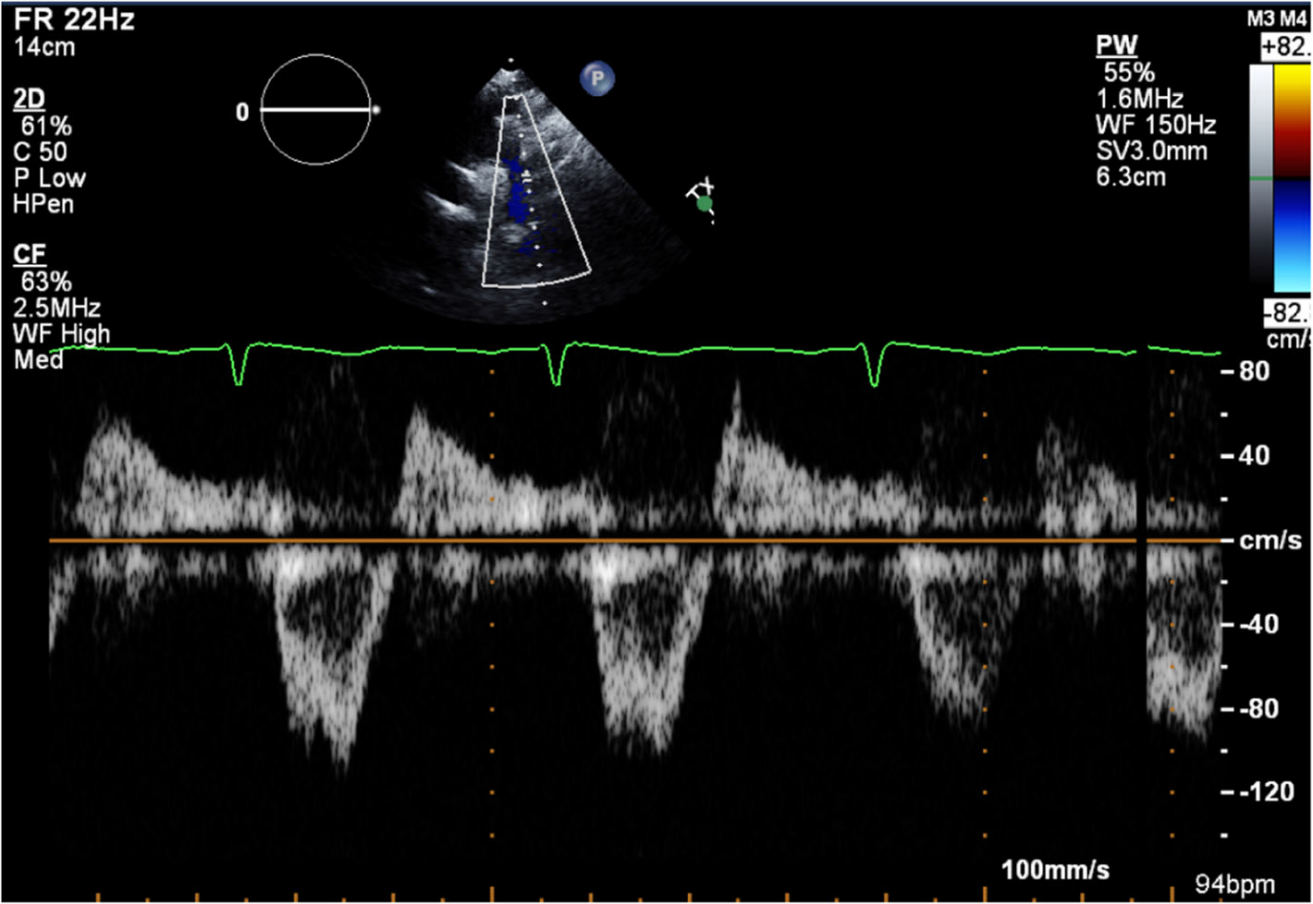
Transthoracic echocardiography suprasternal view with pulsed Doppler echocardiography demonstrating diastolic flow reversal in the descending thoracic aorta without evidence of aortic regurgitation

Transthoracic echocardiography (TTE) was subsequently performed for investigation of the new continuous murmur – other conditions apart from an SVA which can present with continuous murmurs include; patent ductus arteriosus, coarctation of the aorta, coronary arteriovenous fistulas and an aortopulmonary window. TTE showed normal left and right ventricular size and systolic function. To the unwary eye, the colour flow at the RV inflow view may appear like tricuspid regurgitation. However, the continuous wave Doppler clearly demonstrated a continuous wave form throughout systole and diastole (Fig. 1, Videos 1 and 2). Additionally, a significant left-to-right shunt was detected on the colour Doppler between the right coronary sinus and the right atrium (Fig. 2, Video 3). Interestingly, Doppler echocardiography detected diastolic flow reversal in the thoracic descending aorta which is more commonly seen in severe aortic regurgitation (Fig. 3).

**Fig. 4:**
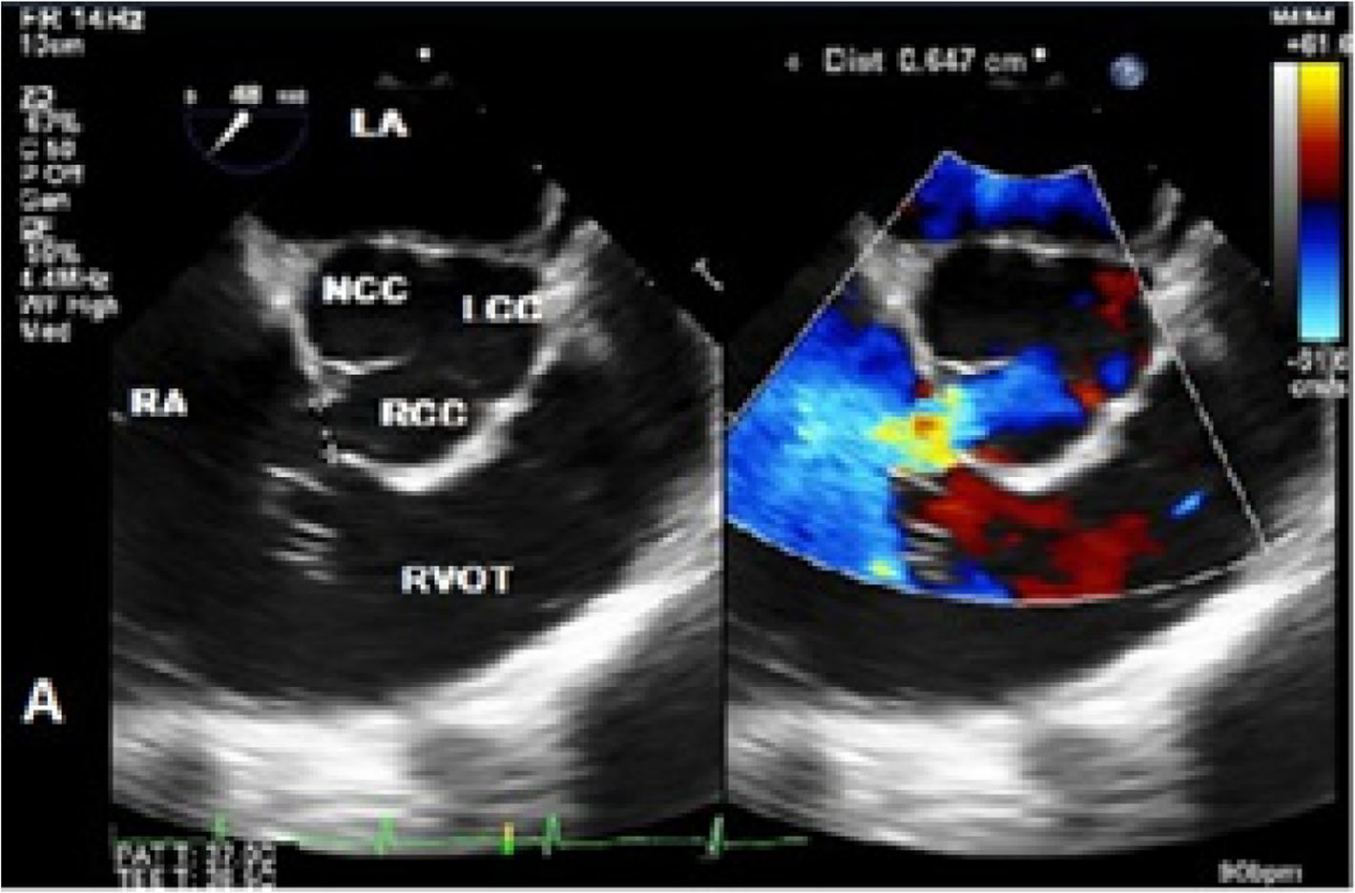
Two-dimensional transoesophageal echocardiography in mid-oesophagus short axis with colour compare view showing a left-to-right shunt from the right sinus of Valsalva to the right atrium. RVOT, right ventricular outflow tract; RV, right ventricle; RA, right atrium; LA, left atrium; RCC, right coronary cusp; NCC, non-coronary cusp; LCC, left coronary cusp

The patient then underwent transoesophageal echocardiography (TOE) which showed a rupture of the right sinus of Valsalva aneurysm with shunting into the right atrium (Fig. 4, Video 4). The left-to-right shunt in the right atrium was directed towards the centre of the tricuspid valve, imitating significant tricuspid regurgitation. The tricuspid valve itself was normal in structure and function. A small saccular aneurysm of the right coronary sinus was seen extending into the junction between the right atrium and ventricle. A trileaflet aortic valve was noted with no regurgitation. There were no associated atrial or ventricular septal defects.

**Fig. 5:**
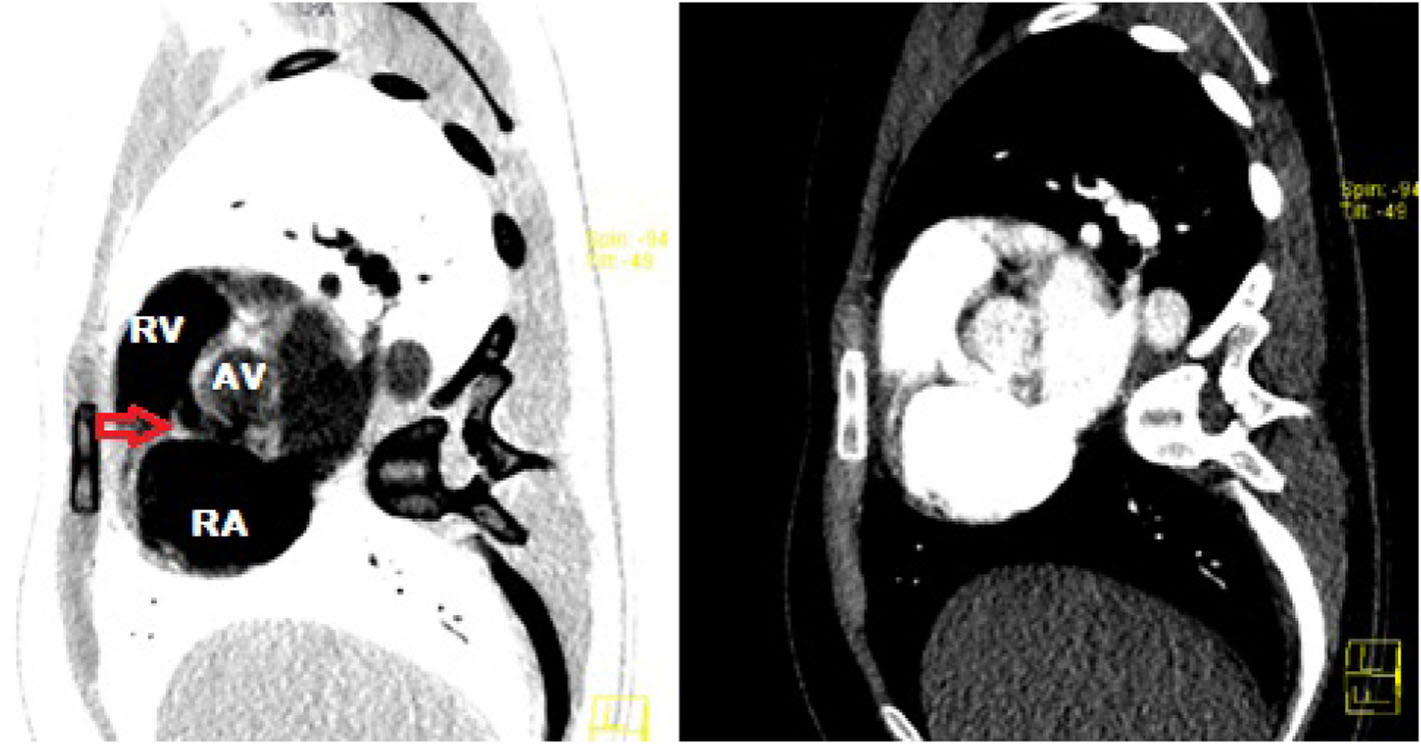
Reconstructed computed tomography pulmonary angiography sagittal view image shows intravenous contrast in the communication between the aortic root and probable right ventricle (red arrow). RV, right ventricle; RA, right atrium; AV, aortic valve

CTPA images were retrospectively reconstructed which visualised the SVA and shunt, likely originating from the right coronary sinus into the right ventricle (Fig. 5).

**Fig. 6:**
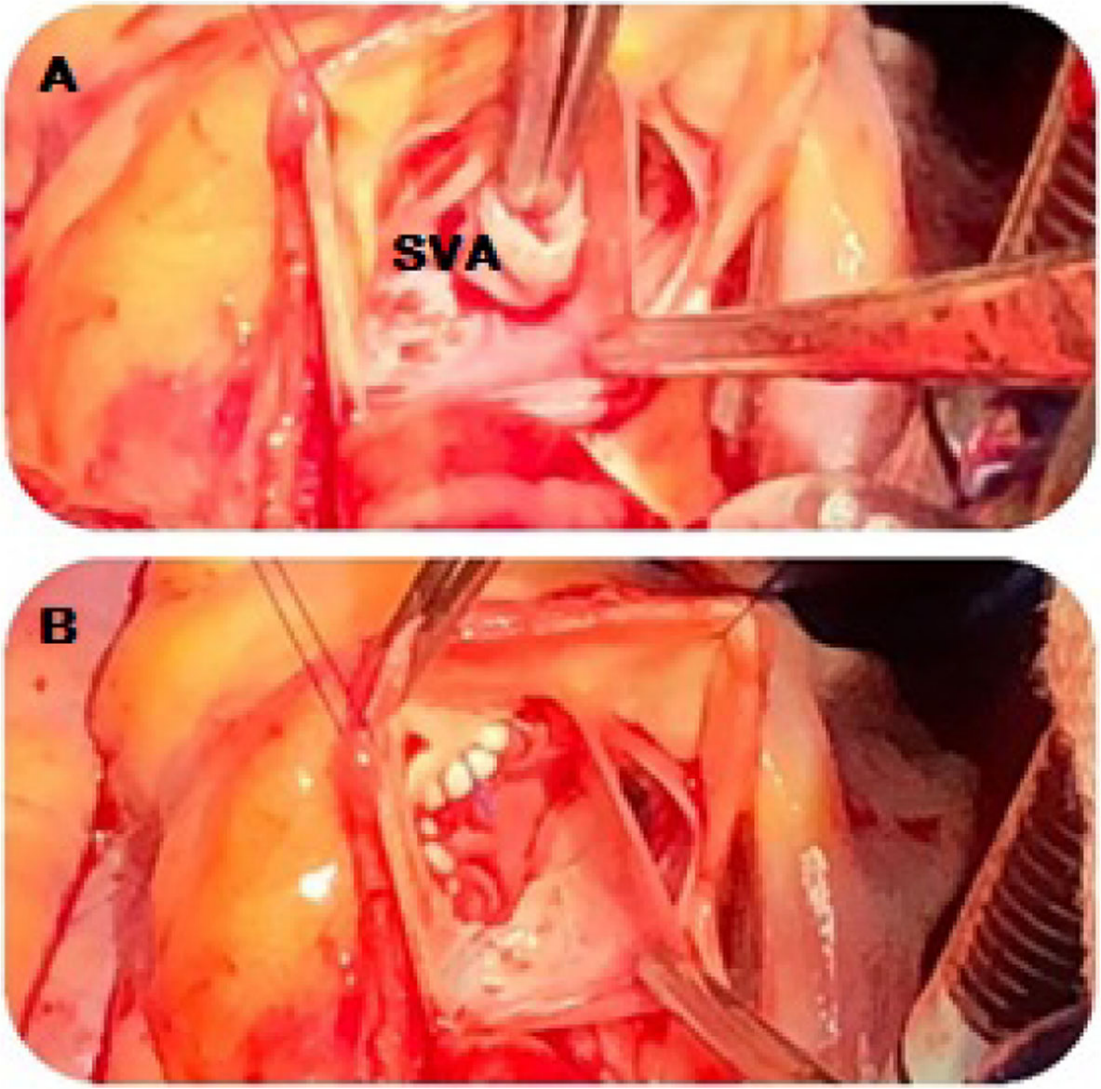
Intraoperative view; **a** Right sinus of Valsalva aneurysm and fistula. **b** Repair of aneurysm. SVA, sinus of Valsalva aneurysm

Since the patient had an acutely ruptured SVA, a surgical repair was proposed given significant left-to-right shunting and to prevent RV overload. She underwent open-heart surgery with excision of the aneurysm and pericardial patch repair of the right coronary cusp-SVA fistula (Fig. 6). At routine outpatient follow-up 4 weeks later, she reported no further symptoms, resolution of her cardiac murmur and successful repair on repeat transthoracic echocardiography.

## Discussion and conclusions

### A rare cardiac anomaly

The diagnosis of a sinus of Valsalva aneurysm can be challenging. In our case, the continuous machinery murmur was underappreciated at admission and an alternative diagnosis (pulmonary embolism) was considered and treated with full dose anticoagulation - which could be detrimental in some cases. Routine CTPA examination did not initially demonstrate the lesion (as it was not a dedicated cardiac CT scan protocol) until further reconstruction was performed in retrospect. This demonstrates the importance of meticulous physical examination technique and identification of a significant cardiac murmur, with recognition of the need for urgent echocardiography to facilitate timely diagnosis.

A congenital SVA is frequently associated with Marfan syndrome, Ehlers-Danlos syndrome, or other connective tissue disorders [1–3]. Acquired aneurysms have been associated with trauma, atherosclerosis, infective endocarditis, iatrogenic injury during aortic valve replacement, syphilis and collagen vascular disorders [1–3]. This patient was considered to have a congenital form (although unproven) given the lack of physical findings of a connective tissue disorder, family history and negative connective tissue disease screening. Genetic testing was discussed but not performed due to financial concerns given her current residency status.

Congenital SVAs are usually associated with other cardiac anomalies, including ventricular septal defect, aortic valve dysfunction, bicuspid aortic valve, mitral and tricuspid regurgitation, and coarctation of aorta [3–5]. Other uncommon associated cardiac abnormalities include Ebstein’s anomaly, transposition of the great arteries or a patent ductus arteriosus [3–5].

SVAs are frequently asymptomatic and consequently seldom diagnosed. They commonly arise from the right coronary sinus (in 70% of cases), and the non-coronary sinus in 25% if rupture occurs, a shunt more commonly develops into the right ventricle or right atrium [4]. Cases of right ventricular outflow obstruction, coronary artery compression with infarction, conduction disturbances, endocarditis and thrombus within the aneurysmal cavity have also been reported [6].

The most frequent complication of an SVA is rupture. Rarely, rupture is into the left atrium, left ventricle, pericardial cavity or pulmonary artery. Aneurysm rupture usually occurs between 20 and 40 years of age but cases of rupture in infancy and octogenarians have been reported [4]. In case of rupture, patients mostly present with a gradual onset of symptoms including chest pain, dyspnoea, palpitations, fatigue and syncope [4, 6].

### Diagnostic modalities

Several imaging modalities are available to diagnose an SVA with or without rupture. Traditionally echocardiography, either transthoracic or transoesophageal, has been the first-line imaging technique. Other imaging tools include multi-slice computed tomography (MSCT) and magnetic resonance imaging [7].

Echocardiographic evaluation with colour Doppler will reveal continuous flow in systole and diastole in a ruptured SVA in about 70% of cases [8]; therefore TOE has become the gold standard imaging modality to precisely define the anatomy, and any associated cardiac anomalies [8].

Limitations of TTE were present in our case, in which the exact path of the shunt was difficult to demonstrate whereas TOE was very accurate for establishing a diagnosis. Additionally, TTE was suspicious for tricuspid regurgitation whereas TOE confirmed normal tricuspid valve anatomy and function. The demonstrated diastolic flow reversal in the descending thoracic aorta occurs due to the significant left-to-right shunt from the aortic root to the right-sided cardiac chamber creating similar haemodynamic physiology as severe aortic regurgitation.

### Management

Asymptomatic unruptured SVA has an unknown natural history and optimal management is unclear [3]. Surgical intervention is warranted once an unruptured aneurysm becomes symptomatic with the development of malignant arrhythmias, ostial coronary artery occlusion or right ventricular outflow tract obstruction [3]. Ruptured SVAs require early surgical intervention since median survival is 3.9 years if untreated [9]. Death is usually due to congestive heart failure [4].

Percutaneous transcatheter closure of an SVA has been reported using different occlusive devices which obviates the need for an open-heart surgery. Experience in this field is growing but large clinical trials with long-term follow-up are lacking [2, 10].

Surgical treatment technique varies based on the size of the aneurysm, presence of an aortic valve abnormality, and other concurrent cardiac anomalies [2]. These procedures include primary closure, patch repair or aortic root replacement with or without valve replacement. Peri-operative mortality is between 1.9 to 3.9% [4, 6]. After repair, patients’ life-expectancy approximates that of the healthy population. Satisfactory prognosis after surgical repair has been reported with approximately 90% event-free survival at 15 years [6, 11].

### Limitations

This patient presented to a relatively small regional hospital (250-bed hospital) with limited resources - overnight CT scanning and echocardiography are only available for life threatening conditions. Cardiac MRI and cardiac CT are not available in this centre.

In conclusion, rupture of a sinus of Valsalva aneurysm, though rare, must be considered in a patient with sudden onset of shortness of breath, chest pain and palpitations in the presence of a continuous loud murmur on examination. Echocardiography is the preferred imaging modality to confirm the diagnosis. Early intervention is indicated.

### Learning points

- Sinus of Valsalva aneurysms can be asymptomatic until acute rupture. Rupture most commonly occurs into either the right atrium or right ventricle (rarely both).
- Physical examination is invaluable in patient assessment - a new continuous murmur is always significant and must prompt urgent echocardiography to facilitate timely diagnosis and treatment.
- Transthoracic and transoesophageal echocardiography are both excellent imaging modalities to investigate sinus of Valsalva aneurysms and associated cardiac abnormalities.

## Supplementary information

**Additional file 1: Video 1.** Two-dimensional transthoracic echocardiography in parasternal long axis right ventricular inflow view showing a tricuspid valve with a mobile filamentous structure extending through it.

**Additional file 2: Video 2.** Two-dimensional transthoracic echocardiography with colour Doppler in parasternal long axis right ventricular inflow view showing regurgitation jet through tricuspid valve.

**Additional file 3: Video 3.** Two-dimensional transthoracic echocardiography with and without colour Doppler in parasternal short axis view demonstrating a sinus of Valsalva aneurysm and fistula to the right atrium.

**Additional file 4: Video 4.** Two-dimensional transoesophageal echocardiography with and without colour Doppler showing a right coronary cusp sinus of Valsalva aneurysm with a fistula in to the right atrium.

## Data Availability

Not applicable.

## Abbreviations

CT scan: Computed tomography scan
CTPA: Computed tomography pulmonary angiography
MRI: Magnetic resonance imaging
SVA: Sinus of Valsalva aneurysm
TOE: Transoesophageal echocardiography
TTE: Transthoracic echocardiography
